# Magnetic Resonance Imaging for tracking cellular patterns obtained by Laser-Assisted Bioprinting

**DOI:** 10.1038/s41598-018-34226-9

**Published:** 2018-10-25

**Authors:** Olivia Kérourédan, Emeline Julie Ribot, Jean-Christophe Fricain, Raphaël Devillard, Sylvain Miraux

**Affiliations:** 1 INSERM, Bioingénierie Tissulaire, U1026, F-33076 Bordeaux, France; 2 CHU de Bordeaux, Services d’Odontologie et de Santé Buccale, F-33076 Bordeaux, France; 3 Centre de Résonance Magnétique des Systèmes Biologiques, UMR5536, CNRS/Univ. Bordeaux, F-33076 Bordeaux, France; 4 ART BioPrint, INSERM, U1026, F-33076 Bordeaux, France

## Abstract

Recent advances in the field of Tissue Engineering allowed to control the three-dimensional organization of engineered constructs. Cell pattern imaging and *in vivo* follow-up remain a major hurdle in *in situ* bioprinting onto deep tissues. Magnetic Resonance Imaging (MRI) associated with Micron-sized superParamagnetic Iron Oxide (MPIO) particles constitutes a non-invasive method for tracking cells *in vivo*. To date, no studies have utilized Cellular MRI as a tool to follow cell patterns obtained via bioprinting technologies. Laser-Assisted Bioprinting (LAB) has been increasingly recognized as a new and exciting addition to the bioprinting’s arsenal, due to its rapidity, precision and ability to print viable cells. This non-contact technology has been successfully used in recent *in vivo* applications. The aim of this study was to assess the methodology of tracking MPIO-labeled stem cells using MRI after organizing them by Laser-Assisted Bioprinting. Optimal MPIO concentrations for tracking bioprinted cells were determined. Accuracy of printed patterns was compared using MRI and confocal microscopy. Cell densities within the patterns and MRI signals were correlated. MRI enabled to detect cell patterns after *in situ* bioprinting onto a mouse calvarial defect. Results demonstrate that MRI combined with MPIO cell labeling is a valuable technique to track bioprinted cells *in vitro* and in animal models.

## Introduction

During the last decades, regenerative medicine has benefited from innovative approaches related to tissue engineering. Among novel technological strategies, cell bioprinting has emerged as a promising tool to develop biological substitutes that allows accurate reproduction of a complex three-dimensional tissue architecture and cell microenvironment, including cell-cell and cell-microenvironment interactions^1,2^. Bioprinting is currently defined as “computer-aided, automatic, layer-by-layer deposition, transfer and patterning of biologically relevant materials”^1,3^. One of the main advantages of bioprinting is its ability to control structure and functional properties of fabricated tissue-like structures^4^. Laser-Assisted Bioprinting (LAB) is an exciting new addition to the bioprinting arsenal that traditionally consisted of inkjet and extrusion-based methods. Combined with other additive manufacturing process, LAB has significant potential for applications in Tissue Engineering due to its ability to create two- or three-dimensional constructs with desired resolution and organization^5^. LAB has been successfully used to print a large variety of biological components such as hydrogels, DNA, peptides and live cells^6–9^. This technology provides significant advantages such as rapidity, reproducibility, precision, high cell viability and density^4,5,10^. Because it employs a nozzle-free approach, LAB is able to overcome multiple issues related to the orifice clogging, non-reproducibility due to solution viscosity and cross-contamination, which are common among other bioprinting techniques. Moreover, as a non-contact technology, LAB has shown promise for *in vivo* computer-assisted medical interventions and *in situ* tissue engineering applications, where other bioprinting strategies may not work. Indeed, bioprinting is usually reported in the literature for *in vitro* or *ex vivo* experiments^11,12^, or for *in situ* bioprinting during relatively non-invasive surgical procedures such as skin regeneration^13^. In contrast, LAB has been used, as a proof of concept, to print particles of nanohydroxyapatite, *in situ*, onto a mouse calvaria defect^14^. Further, *in situ* bioprinting of biological components and mesenchymal stromal cells has been utilized to assess the impact of different geometric cell patterning, obtained by LAB, on bone regeneration *in vivo*^15^. To the best of our knowledge, these studies are the first experiments that demonstrated successful application of any bioprinting technology for *in situ* patterning in a context of bone regeneration. More complex structures like cardiac patches have been designed by LAB; however, that process involved two separate steps: *in vitro* creation of the patch followed by *in vivo* implantation^16^.

Combination of bioprinting technologies with stem cell biology has become widespread in regenerative medicine. Among isolated stem cell populations, dental stem cells have many advantages, including their accessibility, capacity for self-renewal, potential for multi-differentiation and possible autologous implantation. Several *in vivo* studies demonstrated regeneration of bone and neural tissue following implantation of dental tissue-derived stem cells^17–19^. For example, Stem Cells from the Apical Papilla (SCAP) can differentiate into osteogenic, adipogenic, chondrogenic, and neurogenic lineages under inductive conditions *in vitro*^20^. These stem cells have strong potential for regeneration of several tissues and are becoming increasingly important in both regenerative dentistry and medicine. Therefore, *in situ* bioprinting of dental stem cells is a promising approach in tissue engineering, especially for bone regeneration.

*In situ* bioprinting onto deeper tissues, such as bone, is associated with difficulties in cell pattern imaging and *in vivo* follow-up. However, for the successful application of this technology it is crucial to track printed cells *in vivo* in a non-invasive manner, in order to check the quality of printed patterns immediately after the bioprinting process, to study their persistence and evolution over time, and to provide insight into cellular proliferation and migration dynamics^21^. To date, no technology has been able to achieve this.

Magnetic Resonance Imaging (MRI) is a non-invasive and non-irradiative imaging technique that allows performing longitudinal studies and repetitive scans without harmful effects. It also enables gathering information over the entire depth of a patient’s or an animal’s body. In order to specifically detect and track bioprinted cells, Cellular MRI can be employed. Gadolinium ions need to be chelated to decrease their cytotoxicity, limiting their internalization by cells^22^. Mn-based contrast agents are very powerful T1 contrast agents, but their cytotoxicity restrains their use^23^. Fluorine-based contrast agents are highly specific but, due to a low sensitivity, a high amount of Fluorine atoms have to be present within the cell of interest^24^. Thus, this type of labeling may be incompatible with some cell types that have low labeling abilities. On the contrary, superparamagnetic particles, mostly based on iron oxides, are efficiently internalized by many cell types. Consequently, this labeling is the most commonly used in Cellular MRI. Among the range of commercially available T2 contrast agents, Micron-sized Iron Oxide Particles (MPIO) contain the highest amount of iron oxide cores, which maximizes the sensitivity of detection of the labeled cells on standard T2 and T2*-weighted MR images. These particles have been used in preclinical studies as they are non-biodegradable, enabling a long follow-up of the labeled cells^25–27^. MPIO have already been used to label mesenchymal stem cells (MSCs). It has been shown that they do not induce any cytotoxicity or differentiation^28–31^. Nevertheless, to our knowledge, no studies have considered cell labeling with MPIO in order to follow cell patterns organized using bioprinting technologies.

Therefore, the objective of this work was to combine Laser-Assisted Bioprinting with the methodology of tracking MPIO-labeled stem cells using MRI. Optimal MPIO concentrations for tracking *in vitro* and *in vivo* bioprinted cells were determined. The precision of the printed patterns was compared using MRI and confocal microscopy. Cell densities within the patterns and the corresponding MRI signals were also evaluated. MRI was then used to track cell patterns after *in situ* bioprinting onto a mouse calvaria bone defect model.

## Results

### Determination of the optimal MPIO concentration for tracking bioprinted cell patterns using MRI *in vitro*

**Figure 1:**
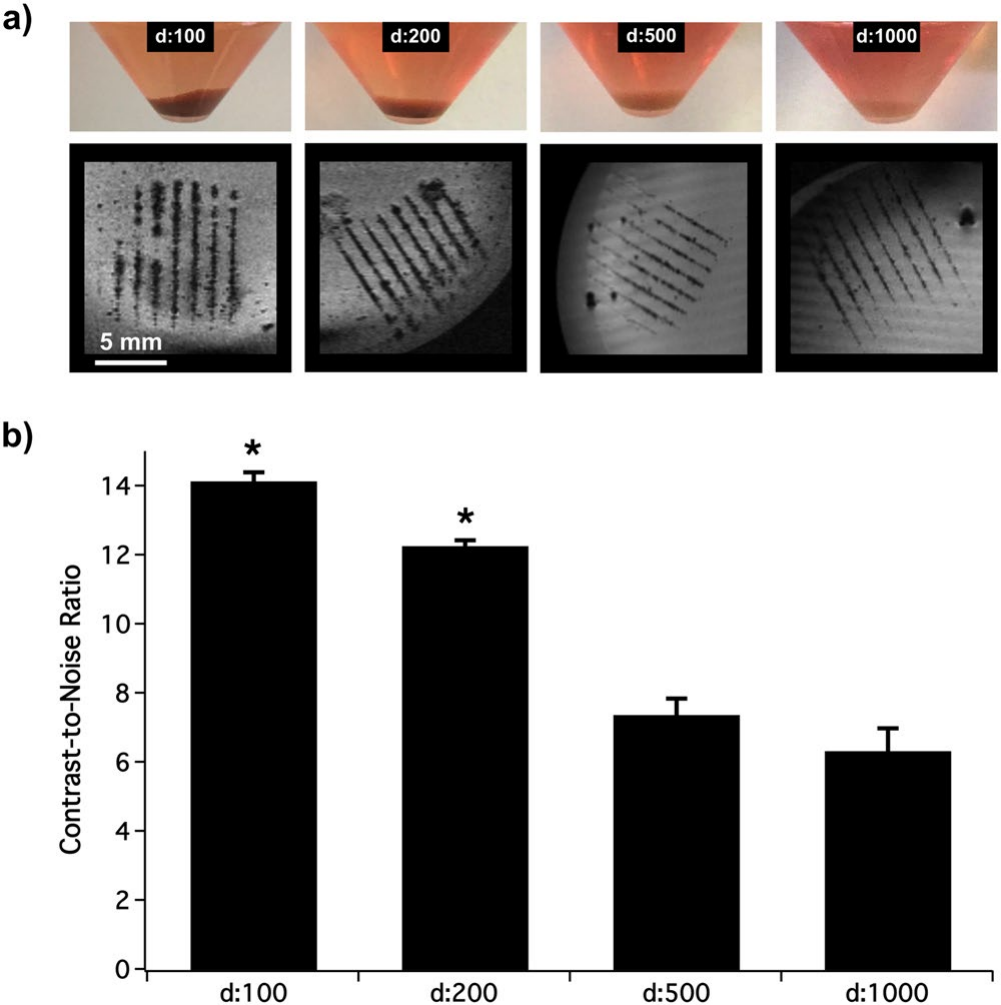
Determination of the optimal MPIO concentration for cell incubation. (**a**) Pictures of MSC pellets after their incubation with decreasing concentrations of MPIO (d:100, d:200, d:500 and d:1000 from the stock solution at 4.5 mgFe/mL) and the corresponding MR T2*-weighted image (spatial resolution: 137 × 137 × 141 µm) after their bio-printing in a line pattern. (**b**) Graph showing the Contrast-to-Noise Ratio (CNR) of the lines with background measured on T2*-weighted images in function of the MPIO dilution factor. *means significantly different with d:500 and d:1000.

In order to determine the optimal conditions to obtain sufficient MRI signal intensity for tracking cell patterns *in vitro*, cells were incubated with different concentrations of MPIO (1:1000, 1:500, 1:200, 1:100 from the stock solution containing 4.5 mg Fe/mL). The bioprinting procedure of the MPIO-labeled cells required a slight increase in the minimal energy (+1 μJ), but otherwise was similar to that for the unlabeled cells. Immediately after printing, cell lines could be identified using T2*-weighted MRI (Fig. 1a). MRI Contrast-to-Noise ratio of the labeled cells significantly decreased when the MPIO dilution was higher than 1:500. A MPIO dilution of 1:200 was chosen as the optimal concentration because it led to a good sensitivity of detection on the MR images and to the most accurate reproduction of printed patterns (Fig. 1b). A higher MPIO concentration (1:100) resulted in less accurate printed patterns with multiple satellites onto the substrate.

### Correlation between pattern cell density and MRI signal *in vitro*

**Figure 2:**
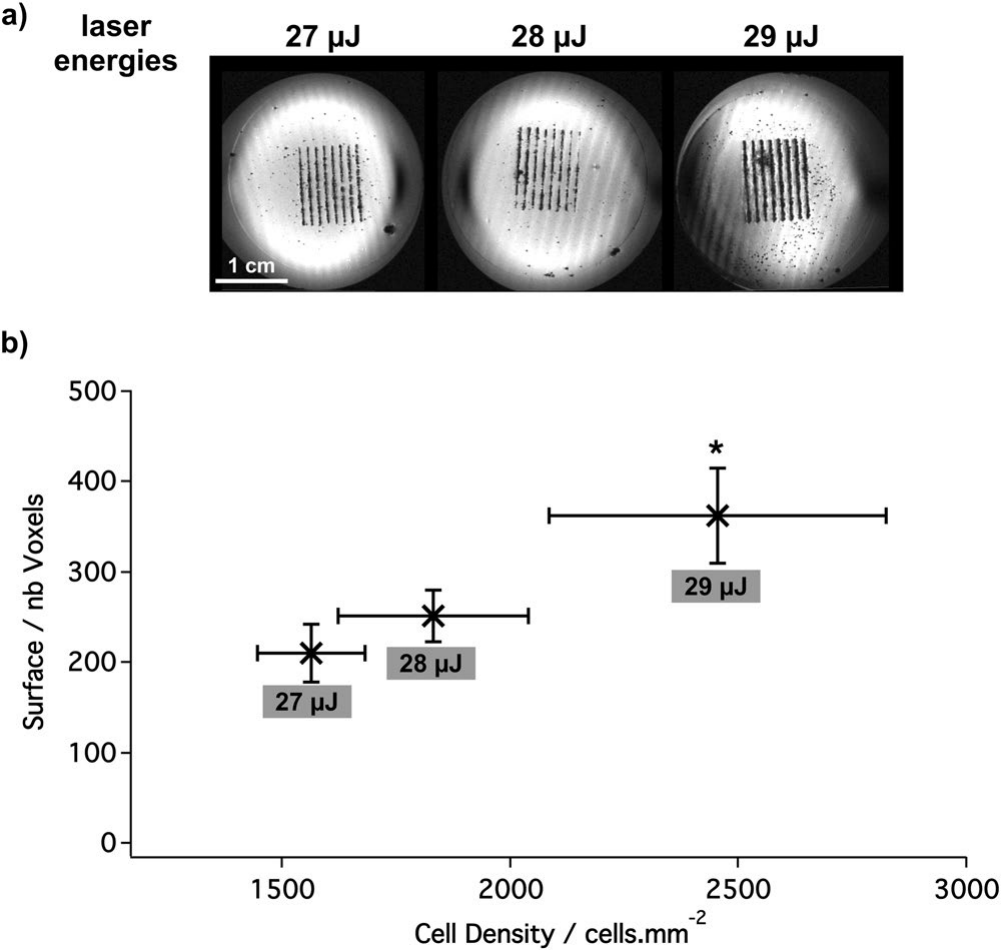
Determination of the optimal cell density. (**a**) MR T2*-weighted images of the line pattern (spatial resolution: 137 × 137 × 52 µm) after the bioprinting of MPIO-labeled MSC with increasing density (laser energy of 27 µJ, 28 µJ and 29 µJ). (**b**) Quantification of the above MR images through the measurement of the amount of voxels per line in function of the density.

Patterns printed with increasing cell densities obtained by varying laser energy were correlated with MRI signal *in vitro* (Fig. 2a). As expected, the thickness of the lines increased with the laser energy. As the density of the bioprinted cells increased from 1562 ± 118 to 1829 ± 209 and 2453 ± 371 cells/mm2, the area covered by the MPIO-labeled cells on the MR images increased from 210 ± 32 to 252 ± 29 and 363 ± 53 voxels, respectively. There was a significant linear correlation was found between the area of the hypo-intense signal and the amount of bioprinted cells (R2 = 0.9991) (Fig. 2b).

### Visualization of cell patterns using MRI and confocal microscopy *in vitro*

**Figure 3:**
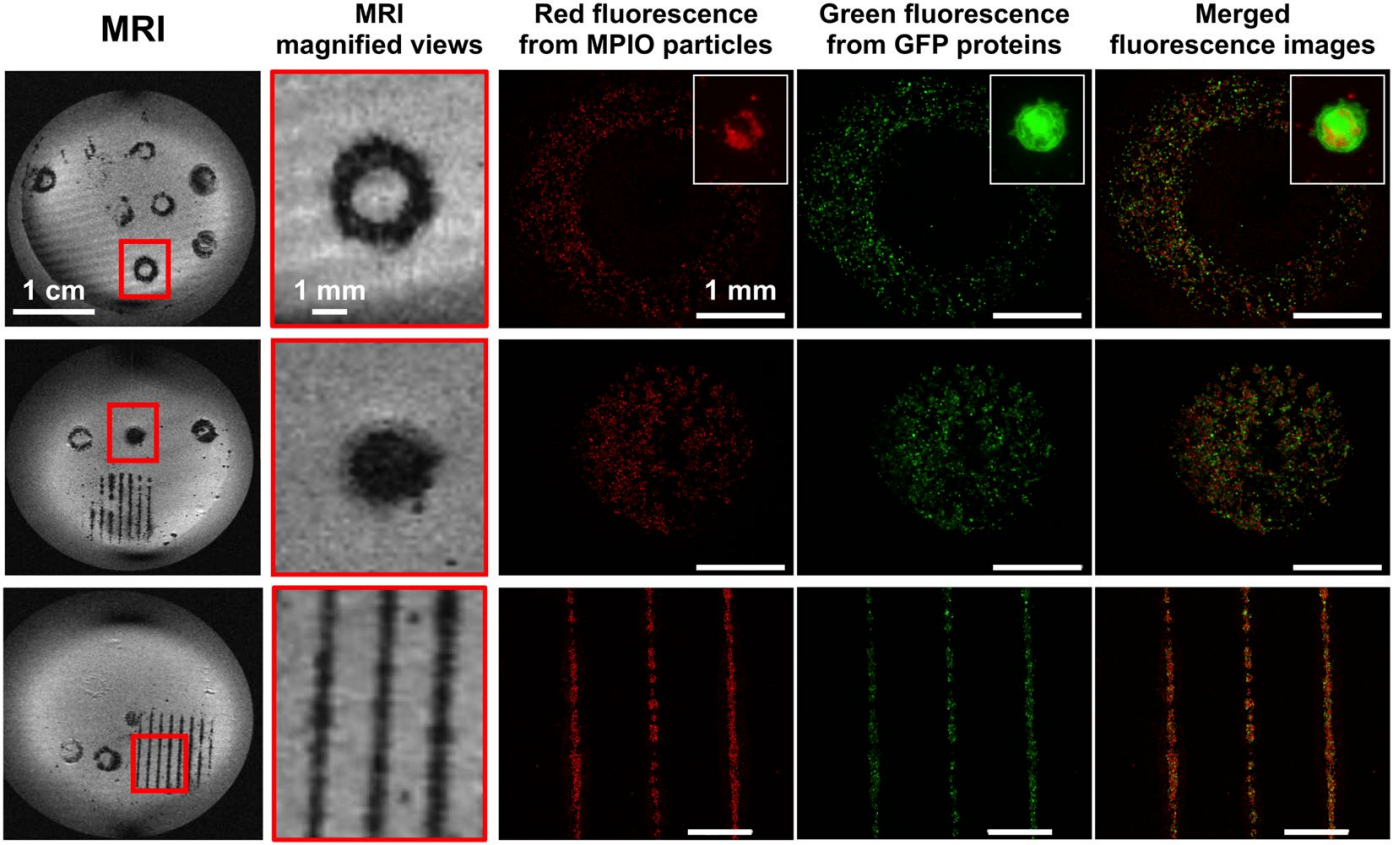
MR and fluorescence detection of three different patterns of MPIO- and GFP- labeled MSC immediately post-printing *in vitro*. The two left columns show MR T2*-weighted images of three different printed patterns and their magnifications (spatial resolution: 137 × 137 × 52 µm). The last three columns show fluorescence microscopy images of the corresponding printed patterns, with respectively FlashRed-MPIO signal, GFP signal, and the merged images. The white inserts show the high magnification view of a GFP- and MPIO-labeled cell.

Confocal microscopy imaging of overlapping green (GFP-cells) and red (Flash Red-MPIO) fluorescence showed that MPIO particles were highly internalized within the MSC cells (inserts). In order to determine the optimal cell patterns for use in the subsequent *in vivo* studies, three different printed designs were tracked: a ring, a disk and parallel lines. The accuracy of the printed patterns was determined by comparing MRI and confocal microscopy images (Fig. 3). The results demonstrated that the MR images accurately reproduced the overall shape of the printed patterns, and that the lines of less than 100 micrometers wide were easily detectable using MRI. The Supplementary Fig. 1 shows that even if some MPIO are detectable at the center of the ring by fluorescence, no signal is detected on the corresponding MR image.

### *In vitro* follow-up of the cell patterns using MRI

**Figure 4:**
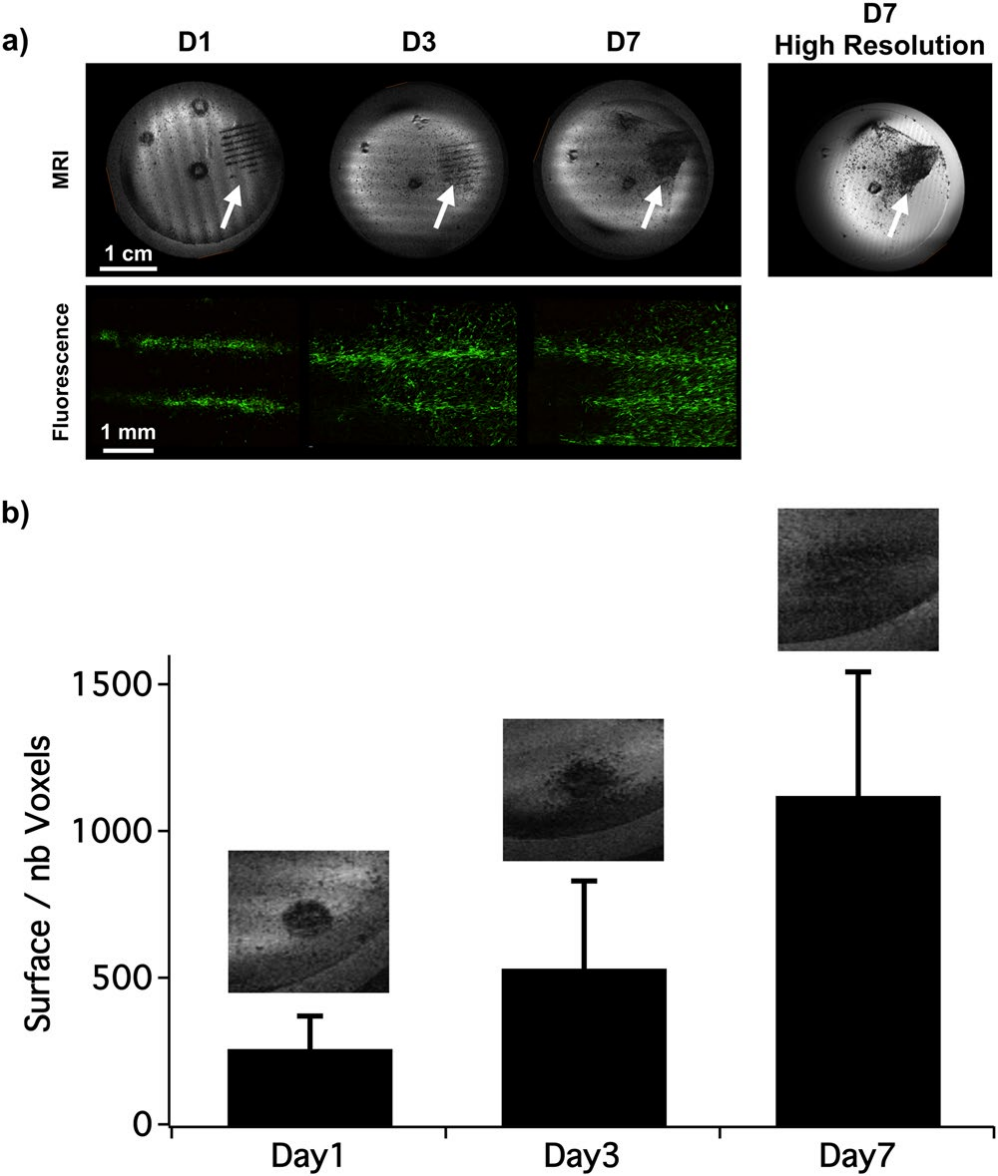
*In vitro* longitudinal MR follow-up of the bioprinted cells. (**a**) MR T2*-weighted images (spatial resolution: 137 × 137 × 52 µm) acquired at day 1 (D1), 3 (D3) and 7 (D7) of the same live MPIO-labeled MSC bio-printed in a line pattern (arrows). The corresponding magnified GFP-fluorescence images are shown below. The far right MR image was acquired at D7 but with longer acquisition time. (**b**) Quantification of the surface covered by the labeled MSC over time after their bio-printing in a 2 mm-disk (the corresponding magnified MR T2*-weighted images are shown on the top of each graph bar).

In order to determine if an *in vivo* longitudinal follow-up could be achieved, a 7-day *in vitro* follow-up of live MPIO-labeled MSC was performed (Fig. 4a). During this time period, cells were proliferating (1 doubling per 2 days) and a progressive disorganization of patterns was observed similar to that in monoculture^32^. The MR images acquired with a short scan time (only 4 min) were nevertheless able to show the spread of the cells within the petri dish. The MPIO-labeled MSC were still easily detectable on the MR images at 7 days in culture. High-resolution MR images were also acquired on fixed cells at different time points after the bioprinting procedure of a disk pattern. For this, identical patterns were bioprinted on multiple petri dishes to enable a longitudinal follow-up with fixed cells. MR imaging of fixed samples at different time points showed that the area covered by the MSC and detected by MRI increased from 262 ± 111 voxels at day 1 to 537 ± 297 voxels at day 3 and 1121 ± 422 voxels at day 7 (Fig. 4b), confirming the spread of the cells over time.

### MRI detection of cells organized by Laser-Assisted Bioprinting in a mouse model of bone calvaria defect

**Figure 5:**
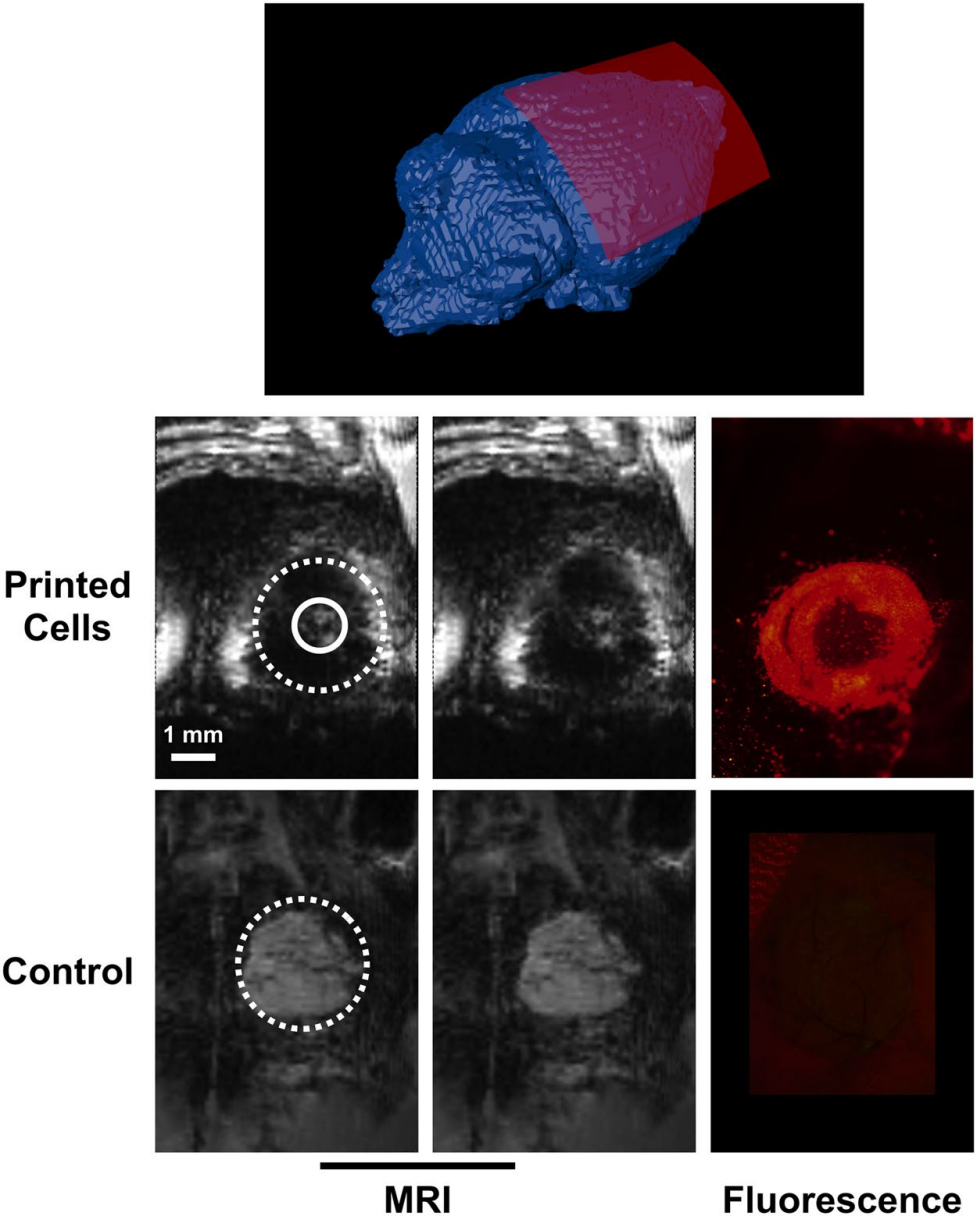
Post-mortem MR and fluorescence images of a mouse calvaria bone defect filled or not with MPIO-labeled cells bio-printed in a ring pattern. A scheme of the position of the reconstructed curved MR slice (red) is shown on top. The left column shows MR curved slice (spatial resolution: 97 × 94 × 94 µm) from mice bioprinted or not (control) with cells. Dashed circles represent the position of the circular bone defect. Labeled cells were bioprinted in a ring pattern between the dashed and plain circles. The middle column is identical to the left one without any indication to better visualize the signal void generated by the labeled cells. The corresponding fluorescence images are also shown on the right column. Scale bar represents 1 mm.

The “ring” pattern was used for *in situ* bioprinting onto a bone calvaria defect in mouse, in order to assess the feasibility of using MRI to track a precise pattern *in vivo*. First, a well-defined ring of printed cells was observed within the bone defect by fluorescence microscopy. Then, high-resolution 3D MRI was performed to image the defect *post mortem*. The MR images showed a ring within the defect with a lack of signal that likely corresponded to MPIO-labeled printed cells. In a control mouse, where the defect had not been filled with bioprinted cells, the entire defect appeared as an area with a homogeneous grey signal on the MR images (Fig. 5).

## Discussion

### This paper reports the first MR images of laser-assisted bioprinted cells labeled with a contrast agent

For this purpose, mesenchymal stem cells were used due to their strong potential for tissue regeneration^33^. In addition, labeling of these cells is particularly efficient with MRI contrast agents, and does not alter neither their proliferation nor their differentiation^34–39^. Nevertheless, it is important to note that the viscosity of the bio-ink was modified when the cells were iron-labeled. This can be due to the higher granularity of the cells because of the MPIO trapped in multiple vesicles within the cells. Increasing the energy of the laser enabled to efficiently deposit the cells on the biopaper and create different patterns *in vitro*. It appears that a compromise is required between cell signal (increasing with MPIO concentration) and resolution of the pattern (decreasing with MPIO concentration).

After bioprinting, MR imaging was performed using T2*-weighted sequences at 7 T in combination with a device adapted to *in vitro* experiments and a dedicated mouse head coil (4 × 4 array) for imaging the mouse head. The loop coil used for the *in vitro* imaging has a very high sensitivity and it enables to image the whole surface of a petri dish. Nevertheless, due to the low amount of liquid present in the petri dish (3 mL representing 3.5 mm high), reference tubes containing water had to be placed under the coil. With such a set-up, images with very high spatial resolution were acquired. In order to recover a high signal to noise ratio, acquisitions of several hours were performed. Even though acquisition of less than 5 min were enough to detect all the specific patterns that were tested, these specific parameters enabled to detect a very small amount of cells and to easily distinguish the geometric bio-printed patterns. In particular, lines of cells of a thickness of less than 200 μm separated by only 1250 μm were detected with a high contrast *in vitro*. These results were confirmed by fluorescence microscopy through the co-localization of the GFP-expressing cells and the FlashRed-MPIO. MPIO are of great importance for this kind of project. Indeed, due to their high sensitivity of detection and the large signal void that they generate^40,41^, single labeled cells have been detected *in vivo*^42,43^. Here, we demonstrated that, a low incubation concentration (4.5 μg/mL) was enough to detect the patterns. Lower concentrations might be considered, but the sequence parameters (especially the TE) would have to be adapted in parallel to increase the sensitivity of detection. Nevertheless, there is a compromise between increasing the T2* sensitivity of the MPIO by lengthening TE and the spatial resolution necessary to detect lines of bio-printed cells. Indeed, in our study, the TE was maintained <7 ms to not generate large signal voids, that could prevent the detection of thin patterns.

In addition, this parameter limited the detection of free MPIO that could have been bioprinted with the labeled cells. As already demonstrated by Dodd *et al*. and Ribot *et al*., iron particles need to be compartmentalized within cells to be detectable^44,45^.

The *in vitro* experiments also demonstrated that, through the measurement of the surface of the hypo-intense signal generated by the MPIO, it was possible to follow the spread of the labeled cells over time, even if they are actively dividing.

In a model of mouse calvaria, the bioprinted MPIO-labeled cells were detected with a very strong MR hypo-intense signal. Therefore, in the case of bioprinting in a smaller defect, even if fewer cells will have to be printed, their detection should be achievable. In addition, the ability to detect the MSC *in vitro* 7 days after their printing suggests a possibility for an *in vivo* follow-up. In this paper, the MR scanning was performed *post-mortem* in order to prevent any motion blurring on the high-resolution MR images and thus optimize the cell detection. Our results suggest, however, that a longitudinal study that wound focus on cell migration, proliferation and bone regeneration is feasible. In order to improve the *in vivo* vascularization, several types of cells can be bioprinted on the same defect. To specifically track each kind of cells, labeling with different contrast agents (para- and superpara- magnetic ones) can be achieved. In that case, T1 and T2*-weighted MR images could be acquired over time^46^. Other existing models obtained using *in situ* bioprinting technology can also benefit from our MRI protocol, in order to localize printed cells onto deep tissues and to investigate cell dynamics *in vivo*. An elegant *ex vivo* model for studying cancer cell behavior has been designed by Burks *et al*. and allowed optical follow-up. With our method, *in vivo* studies can be performed with similar results quality and a more relevant animal model^47^.

In the perspective of translating this methodology to humans, biodegradable contrast agents will have to be employed. Biodegradable MPIO have already been synthesized^48^. Also, fluorine-based contrast agents have been recently used to detect dendritic cells at 3 T in cancer patients^49^. In parallel, laser-assisted bioprinting workstations could be designed to be used inside a sterile operating room and would constitute a new therapeutic approach, personalized and “tailor-made”, allowing for precise organization of cells or biomaterials to implant at a micron scale. In this work, we chose to analyze patterns created by LAB among other bioprinting technologies in order to demonstrate the accuracy of the method. LAB was the ideal technology because the size of droplets generated by LAB is the lowest possible and the positioning of cells is the most precise, compared to other methods^50^. Therefore, ink-jet and extrusion-based bioprinted pattern would also be compatible with MRI follow-up.

One of the limitations of the study is the long acquisition times. To shorten these durations, the TE of the sequence can be lengthened to increase the sensitivity of detection. A bSSFP sequence has been shown to improve the sensitivity of detection of MPIO trapped within cancer cells in the brain when TE/TR were set long compared to Gradient Echo sequence^51^. In addition, using this sequence single labeled macrophage was detected *in vivo* in the mouse brain^52^. This sequence offers the advantage of obtaining 3D images. Nevertheless, the inherent presence of banding artifacts across the images necessitates the acquisition of several images which lengthen the exam duration. Other sequences might be useful to quantify the amount of contrast agents, like a T2 or T2* parametric sequences. Nevertheless, these sequences are usually less sensitive and/or require long scan duration.

In conclusion, the results presented here demonstrate the potential of using MRI and MPIO-labeled cells to track cell patterns organized *in situ* by Laser-Assisted Bioprinting onto mouse calvaria bone defect.

Future work will be focused on *in situ* Laser-Assisted Bioprinting of endothelial progenitor cells in order to assess the role of vascularization on bone regeneration *in vivo*. The results of this study can potentially have an impact on a wide range of applications, such as in wound healing and other conditions where tissue regeneration and the acceleration and enhancement of vascularization are needed. MRI would help achieve a better control of the procedure, monitor the implanted cells and evaluate the healing. Indeed, MRI may be the optimal biomedical imaging technique to visualize specific cells even in deep organs.

## Methods

### Laser-Assisted bioprinting set-up

The LAB workstation used in this study was previously described^53^. Briefly, the laser source consisted on a solid Nd:YAG crystal laser (Navigator I, Newport Spectra Physics, 1064 nm, pulse duration of 30 ns). The scanning system comprised two galvanometric mirrors (SCANgine 14, ScanLab), with a scanning speed of 2000 mm/s. The laser beam was focused on a quartz ribbon that was coated with a thin absorbing layer of gold (60 nm) and a 30 μm layer of cell bioink (donor slide). A dedicated software was used to control pattern design and substrate position. All experiments were performed in air, at room temperature (RT), with a distance of 1000 μm between the ribbon and the substrate. The transfer process was performed using a repetition rate of 1 kHz.

### Substrate preparation

Rat tail collagen type I (Collagen High concentration; Corning, Bedford, MA, USA) was diluted in DMEM to a final concentration of 2 mg/mL. For *in vitro* experiments, 141 μL of this collagen solution was spread at 4 °C onto the quartz substrate. This receiving layer was allowed to solidify for 1 h in controlled atmosphere (5% CO2, 95% RH, 37 °C) prior to perform printing experiments. For *in vivo* experiments, collagen was printed directly to the dura mater of the mouse. In the case of the negative control, without cell printing, bone defect was entirely filled with collagen solution.

### Cell culture

Stem cells from Apical Papilla (SCAPs) were used throughout this study. Cells were isolated from germs of third molars, obtained from young patients at the Service de Chirurgie Buccale du Centre Hospitalier Universitaire de Bordeaux. All experimental protocols were approved (Ministerial approval “DC-2008-412”; Convention INSERM-CHU de Bordeaux). All experiments were performed in accordance with relevant guidelines and regulations. Oral informed consent to have their samples be used for research purposes was obtained from all patients or, if patients were under 18, from a parent and/or legal guardian. Samples were treated anonymously. In order to allow the follow-up of cell upon *in vitro* and *in vivo* printing, SCAPs were transduced with GFP-expressing lentiviral vectors for *in vitro* experiments, and TdTomato-expressing lentiviral vectors for *in vivo* experiments. GFP- and TdTomato-labeled SCAPs were cultured separately in plastic dishes in Minimum Essential Medium alpha (α-MEM, Gibco, Paisley, Scotland, UK) supplemented with 10% fetal bovine serum (FBS, GE Healthcare, Pasching, Austria), in a controlled atmosphere (5% CO2, 95% RH, 37 °C). Passage 6 to 12 were used for the bioprinting experiments.

### Cell labeling with MPIO

SCAPs were labeled with Micron-sized Superparamagnetic iron oxide particles (SuperParaMagnetic Microspheres, Mean Diameter: 0.90 μm, Color: Flash Red, Bangs Laboratories, Fishers, Indiana, USA). Cells were cultured during 24 hours before printing with different concentrations of MPIO (Stock solution containing 1.278 × 10^10^ MPIO/mL (≈4.5 mgFe/mL); incubation of 100 uL/10 mL; 50 uL/10 mL; 20 uL/10 mL and 10 uL/10 mL noted 1:100, 1:200, 1:500 and 1:1000) depending on the experiment. Cells were washed 2–3 times with PBS, harvested using trypsin-EDTA and centrifuged twice at 1000 rpm for 5 min to remove the excess of MPIO that were not internalized by the cells.

### Bioink preparation and printing parameters

SCAPs were detached from the plastic dish with a solution of trypsin. Cells were suspended in α-MEM supplemented with 10% fetal bovine serum. Cell bioink concentration was 70 × 106 cells/mL. A 3 cm diameter quartz slide (Société VM, Epinal, France) was coated with a thin absorbing layer of gold (60 nm) using a sputter coater (EMSCOPE SC500, Elexience). Then, 30 μm-thick film of cell bioink (33 μL) was manually spread on the ribbon surface. The laser focused on the gold layer induces the transfer of droplets of cells onto the collagen-coated substrate according to the selected pattern. In the present study, three geometries of pattern were chosen: successive lines with droplets spaced from 60 μm between spots with 1250 μm between each line of spots, a disk with 2 mm diameter and a ring with external and internal diameter of 3 and 2 mm respectively. Depending on the experiment, laser energies from 27 μJ to 29 μJ were used in order to modify cell densities of printed patterns^54^.

### *In vitro* labeled cells follow-up

A 7-day *in vitro* follow-up of live or fixed MPIO-labeled MSC was performed. To do so, four sets of experiments were started in parallel. The first set included the bioprinting of live cells and low resolution MR scanning at Day 1, 3 and 7. The same petri dish was thus imaged for 4 min and then put back in the incubator until the next MRI session (Fig. 4a). The last three sets of experiments included the bioprinting of live cells, their fixation at Day 1, 3 or 7 and high resolution MR scanning (Fig. 4b).

### Animal procedures

The procedures and mice handling were based on the principles of Laboratory Animal Care formulated by the National Society for Medical Research and approved by the Animal Care and Experiment Committee of University of Bordeaux, Bordeaux, France (Ref. 201701051243776-V2 APAFIS #8442). Experiments were carried out in accredited animal facilities following European recommendations for laboratory animal care (EU Directive 2010/63/EU for animal experiments). Two 10-week-old NOG female mice, weighting 25–26 g (Charles Rivers, France), were used in this work to compare MRI imaging between a mouse with and without bioprinting procedure. The surgical procedure used in this study was previously described^15^. Briefly, mice were anesthetized with a solution of Ketamin (Imalgen, Merial, France) and Xylazin (Rompun, Bayer, France) through intraperitoneal injection. After performing skin antisepsis (Betadine), an incision was made in skull midline and the scalp was dissected to expose the calvaria. One lateral 3.3 mm diameter circular bone defect was achieved using a 3.3 mm diameter trephine (Praxis l’instrumentiste, France). One mouse was used for laser bioprinting procedure while the other was used as negative control. Surgical site was irrigated with Sodium Chloride Solution (Proamp 0.9%, Laboratoire Aguettant, Lyon, France). Then, the mouse was placed inside the LAB workstation for *in situ* printing experiment. For the “negative control” mouse, only collagen solution was used in order to fill the bone calvaria defect. At the end of the experiment, tissues were repositioned and sutured using 3/0 vicryl (Johnson and Johnson, USA). Animals were placed in a warm environment before to be taken back to animal facilities. The mice were sacrificed by cervical dislocation, practiced by qualified staff.

### Post-printing characterization and image analysis

Accuracy of the patterns was analyzed *in vitro* by confocal microscopy (Leica TCS SPE, DMI 4000B, Mannheim, Germany) and *in vivo* by fluorescence microscopy (Leica MZ10 F, Leica Microsystems Ltd, Heerbrugg, Schwitzerland). Cell Counter plugin of image processing software ImageJ (Open source, Public domain) was used to calculate cell density in patterns post-printing with defined areas.

## Magnetic Resonance Imaging

### Magnet and gradient system

**Figure 6:**
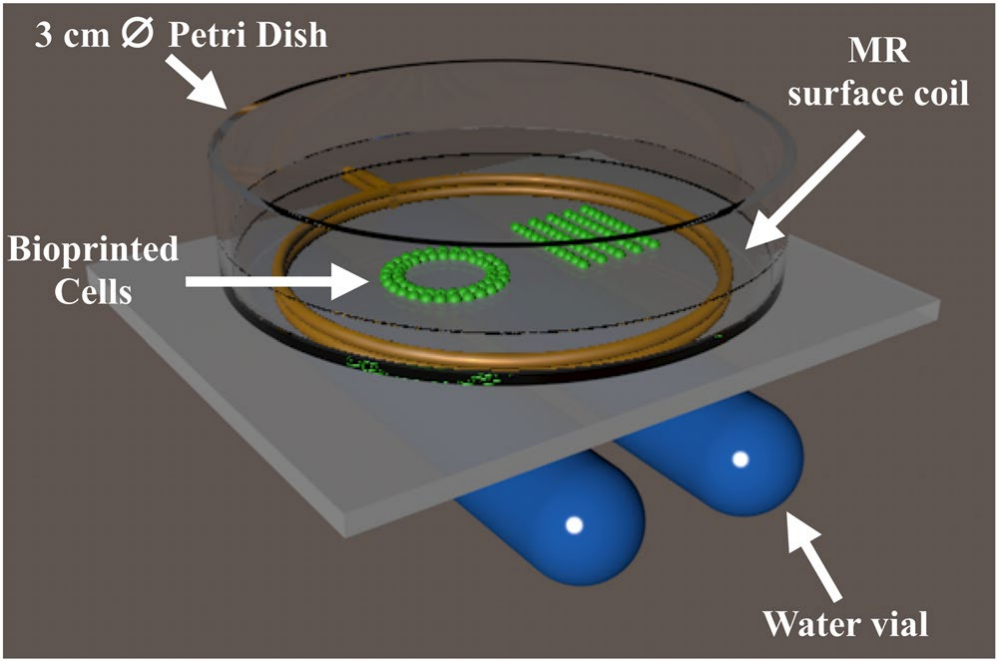
MR set up for the *in vitro* MR imaging of Petri dish containing MPIO-labeled cells bioprinted in different patterns. The coil is represented as the two golden rings, where the petri dish is attached on. Two tubes filled of water are positioned under the coil for the MR adjustments. Bioprinted cells are shown in green and are immersed in cell culture medium.

All experiments were performed on a 4.7 Tesla Bruker Biospec system (Ettlingen, Germany) equipped with a gradient system capable of 660 mT/m maximum strength and 110 μs rise time. For the *in vitro* experiments, a circular surface coil (20 mm diameter, DotyScientific) was used for signal excitation and reception. Two vials fulfilled with water were placed below the coil for MR system adjustment and a thin plate was placed above to precisely installed a Petri dish containing bio-printed cells (Fig. 6).

For the *post mortem* imaging, the animals were positioned within the magnet with the head placed at the center of a 4-element (2 × 2) phased array surface coil (dimensions: 26 × 21 mm2).

### MR sequences

All the MR images were acquired using a T2*-weighted gradient echo sequence. For the detection of each pattern (Fig. 3), the following parameters were used: Echo Time/Repetition Time (TE/TR) = 2.3/6 ms; Field of View (FOV) = 35 × 35 × 5 mm; matrix = 256 × 256 × 96; flip angle = 10°; number of excitations = 64; reception bandwidth = 150 kHz; Acquisition time = 2 h 37 min. For the Density experiment (Fig. 2), the same parameters were used, except: TE/TR = 6.5/13 ms; reception bandwidth = 30 kHz; Acquisition time = 5 h 40 min. For the MPIO incubation concentration dilution experiment (Fig. 1), the following parameters were used: TE/TR = 3.4/7.5 ms; FOV = 35 × 35 × 18 mm; matrix = 256 × 256 × 128; flip angle = 10°; number of excitations = 1; reception bandwidth = 50 kHz; Acquisition time = 4 min 5 s. These parameters were also used for the *in vitro* follow-up experiment on the live cells (Fig. 4a). For the follow-up realized on fixed cells (Fig. 4b), the same parameters as for the Density experiments were used.

For the *post mortem* experiments (Fig. 5), the following parameters were used: TE/TR = 3.7/8 ms; FOV = 25 × 18 × 6 mm; matrix = 256 × 192 × 64; flip angle = 10°; number of excitations = 256; reception bandwidth = 50 kHz; Acquisition time = 7 h.

### MR image analyses

The images were analyzed using the Amira (TGS, San Diego, CA, USA) and IgorPro (Wavemetrics, Lake Oswego, OR) data processing softwares. The mean Contrast-to-Noise ratio (CNR) between 3 lines of labeled cells and background medium was measured for each MPIO concentration used. In addition, the signal areas covered by 3 disks were measured over time and the signal areas covered by at least 7 “line” patterns were measured as a function of the Density. To do so, a semi-automatic segmentation was performed to select pixels containing a signal lower than 3 times the background signal of the culture medium.

### Statistical analysis

Statistical analyses were performed using GraphPad Prism software (GraphPad, San Diego, CA, USA) using a Student t test. Differences were considered to be statistically significant with p < 0.05.

## Electronic supplementary material

Supplementary Figure 1

## Data Availability

All data generated or analysed during this study are included in this published article.
