# Author Correction: Magnetic Resonance Imaging for tracking cellular patterns obtained by Laser-Assisted Bioprinting

**DOI:** 10.1038/s41598-018-35953-9

**Published:** 2018-11-22

**Authors:** Olivia Kérourédan, Emeline Julie Ribot, Jean-Christophe Fricain, Raphaël Devillard, Sylvain Miraux

**Affiliations:** 1 INSERM, Bioingénierie Tissulaire, U1026, F-33076 Bordeaux, France; 2 CHU de Bordeaux, Services d’Odontologie et de Santé Buccale, F-33076 Bordeaux, France; 3 Centre de Résonance Magnétique des Systèmes Biologiques, UMR5536, CNRS/Univ. Bordeaux, F-33076 Bordeaux, France; 4 ART BioPrint, INSERM, U1026, F-33076 Bordeaux, France

Correction to: *Scientific Reports* 10.1038/s41598-018-34226-9, published online 25 October 2018

The original version of this Article contained a typographical error in the spelling of the author Emeline Julie Ribot, which was incorrectly given as Emeline Ribot. This has now been corrected in the PDF and HTML versions of the Article, and in the accompanying Supplementary Information file.

The Author Contributions section now reads:

O.K. and E.J.R. conceived the idea, carried out the experiments, analyzed the data and wrote the manuscript with assistance from all the authors. J.C.F. participated to the experiments and revised the manuscript. R.D. and S.M. participated to the experiments, contributed to discussions of results, coordinated and supervised the overall project. All authors reviewed and approved the final manuscript.

