# Supplementary figures and images for: Magnetic Resonance Imaging for tracking cellular patterns obtained by Laser-Assisted Bioprinting
Source: Sci Rep. 2018 Oct 25;8:15777. doi: 10.1038/s41598-018-34226-9 (PMC6202323; doi:10.1038/s41598-018-34226-9)

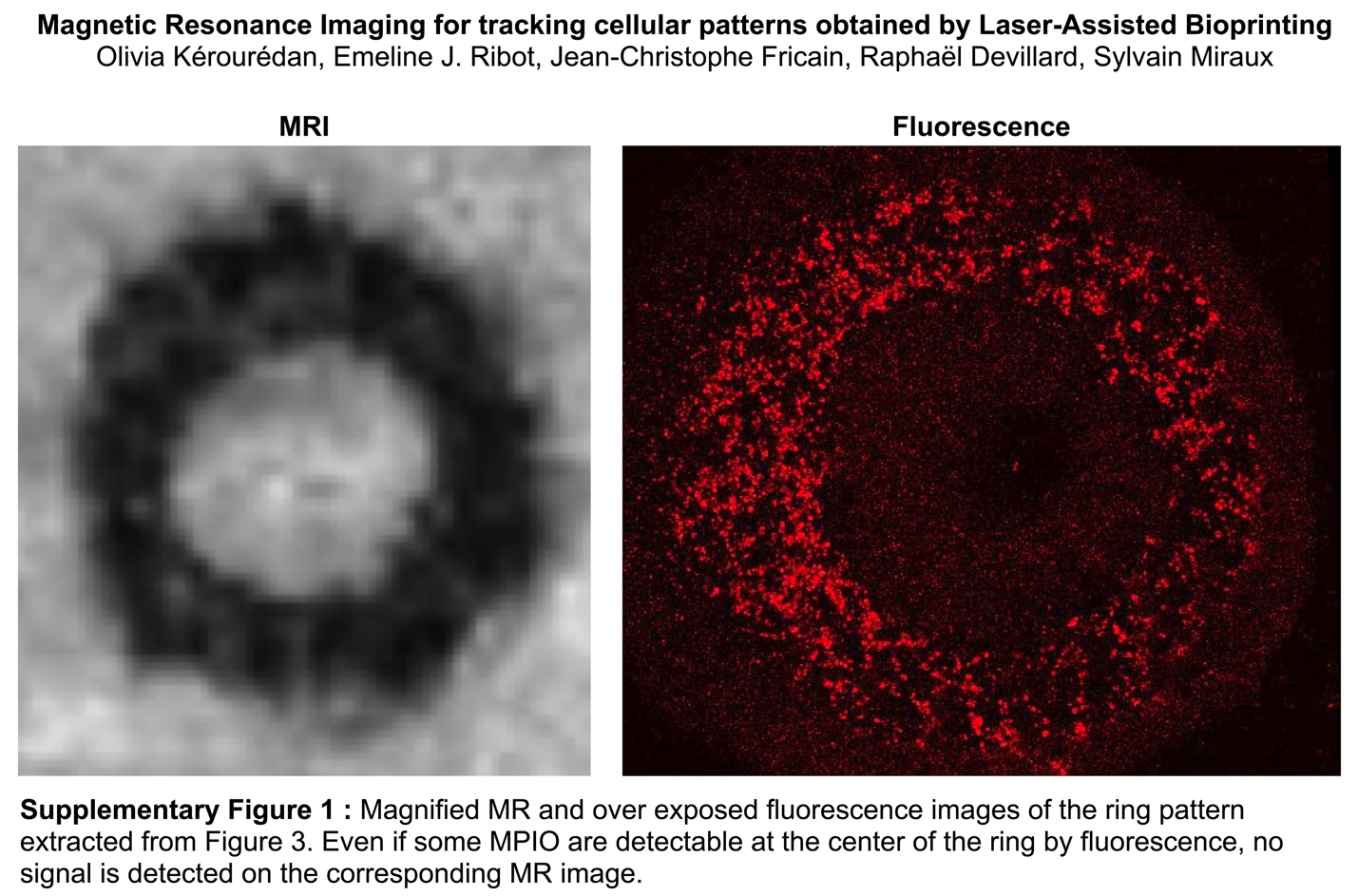

Supplement: Supplementary file 1 — Supplementary Figure 1 [file 41598_2018_34226_MOESM1_ESM.tif]
